# Effect of a cover crop on the aphid incidence is not explained by increased top-down regulation

**DOI:** 10.7717/peerj.13299

**Published:** 2022-05-24

**Authors:** Jeniffer K. Alvarez-Baca, Xiomara Montealegre, Cécile Le Lann, Joan Van Baaren, Blas Lavandero

**Affiliations:** 1 CNRS, ECOBIO (écosystèmes, biodiversité, évolution)—UMR 6553, Université Rennes I, Rennes, Bretagne, France; 2 Laboratorio de Control Biológico, Universidad de Talca, Talca, Maule, Chile

**Keywords:** *Aphidius platensis*, *Myzus persicae*, *Brachycaudus helichrysi*, *Avena sativa*, *Prunus domestica*, Winter refuge, Cover crops, Aphids

## Abstract

**Background:**

Cover crops can be used as a habitat management strategy to enhance the natural enemies and their temporal synchronization with a target pest. We examined the effect of winter oat intercropping within organic plum orchards on the natural enemy abundance and seasonal dynamics on the biological control of plum aphids in spring in Central Chile.

**Methods:**

We compared the incidence and abundance of natural enemies and aphid pests from winter to the end of spring using two treatments: (1) plum trees with an oat cover crop (OCC) and (2) plum trees without a cover crop but with spontaneous vegetation (SV). We hypothesized that cover crops allow the development of winter cereal aphids, promoting the early arrival of natural enemies in spring, resulting in an earlier control of plum aphids.

**Results:**

Winter cereal aphids developed well on the OCC, and as a result, a lower plum aphid incidence in spring was observed when compared to the SV. However, the abundance of natural enemies and the parasitism rates cannot explain the positive impacts of the oat cover crop on the aphid populations as there were no differences between treatments. A potential effect of the oat due to chemical and/or physical stimuli (bottom-up effects) could help to explain these results.

## Introduction

Important regulating ecosystem services such as natural pest control in agroecosystems depend on high levels of biodiversity. Therefore, conservation practices such as those carried out with conservation biological control can increase the effectiveness of pest control by the utilization of natural enemies to reduce their mortality and provide alternative resources through manipulation of the environment (Landis, Wratten & Gurr, 2000; Gurr et al., 2017). Increased plant diversity enhances natural enemy survival and activity by providing food sources (*i.e.,* nectar, pollen and honeydew), overwintering shelter and/or alternative prey/host species, which consequently could result in greater pest control (Landis, Wratten & Gurr, 2000; Pickett, Roltsch & Corbett, 2004; Cardinale et al., 2009; Quijas, Schmid & Balvanera, 2010; Gurr et al., 2017; Gontijo, 2019). Higher natural enemy abundances are expected in agroecosystems with a higher cultivated and spontaneous plant diversity than in more simple agroecosystems (*e.g.*, monocultures) (Rusch, Bommarco & Ekbom, 2016; Begg et al., 2017; Perović et al., 2018). Increasing plant diversity can be achieved by different means: (1) by using intercropping, which consists of cultivating two crops at the same time, such as cereals and leguminous crops (Ben-Issa, Gomez & Gautier, 2017); (2) by adding a ground cover crop in between cultivated trees in orchards (Silva et al., 2010); (3) by adding flower strips within and around the fields (Balzan, Bocci & Moonen, 2016; Hatt et al., 2017); and (4) by allowing the growth of spontaneous vegetation around the target crop plant (Bugg & Waddington, 1994). For the three first cases, the added taxonomic plant diversity is low, with one or a few plant species added, but plant selection is aimed at attracting specific natural enemies that attack the target pest. In the latter case, the taxonomic plant diversity is higher; however, the functional diversity may not be considered in terms of the specific benefit of decreasing the target pest. Spontaneous vegetation has been shown to attract natural enemies (Denys & Tscharntke, 2002). However, positive, neutral, and even negative effects of increased plant diversity on natural enemy populations have been observed in different agricultural systems (Poveda, Gómez & Martínez, 2008; Letourneau et al., 2009) and are context-dependent (Thies & Tscharntke, 1999; Thomson & Hoffmann, 2013; Karp et al., 2018). Additionally, even when the abundance of natural enemies is enhanced, it does not always translate into a greater pest control (Andow, 1991).

Even if the spontaneous vegetation could have positive effects in some instances, it is important to select the right plant diversity (functional diversity) rather than to just increase taxonomic diversity (Gagic et al., 2015; Dainese et al., 2019). For instance, the addition of plants may create reservoirs of pests (Blitzer et al., 2012; Paredes, Cayuela & Campos, 2013) and/or enhance negative interactions among natural enemies such as intra/inter-guild competition, predation (Irvin et al., 2006; Lundgren, Wyckhuys & Desneux, 2009; Blitzer et al., 2012; Paredes, Cayuela & Campos, 2013; Gómez-Marco, Urbaneja & Tena, 2016) and hyperparasitism (Van Nouhuys & Lei, 2004; Schooler, De Barro & Ives, 2011; Jeavons et al., 2022). Primary parasitoids could be hyperparasitized by secondary parasitoids, and/or their mummies could be consumed by generalist predators (Snyder & Ives, 2001), which could reduce the strength of top-down control of pests (Sanders et al., 2011). Therefore, multiple trophic levels need to be considered to improve biological conservation methods. Moreover, studying multiple natural enemy guilds potentially participating in pest control together (Jonsson et al., 2010; Tena et al., 2015) also appears to be crucial to understanding their functional redundancy or complementary nature and may lead to management strategies to reduce negative interactions (Irvin et al., 2006; Lundgren, Wyckhuys & Desneux, 2009; Gómez-Marco, Urbaneja & Tena, 2016).

The synchronization and temporal overlapping of pests and their natural enemies are fundamental for the outcome of natural pest control (Welch & Harwood, 2014). For instance, Van Nouhuys & Lei (2004) showed a better synchronization between the parasitoid *Cotesia melitaearum* Wilkinson (Hymenoptera, Braconidae) and its butterfly host *Melitaea cinxia* L. (Lepidoptera, Nymphalidae) at the beginning of the growing season and at warmer temperatures. This resulted in greater pest control, which has been demonstrated as well for other agricultural systems (Landis & Van Der Werf, 1997; Van Nouhuys & Lei, 2004). By contrast, when synchronization between natural enemies and pests is weak (Landis & Van Der Werf, 1997; Landis, Wratten & Gurr, 2000; Yang et al., 2017), a slight delay in the arrival of natural enemies can lead to a high pest growth rate and to ineffective biological control (Raymond, Ortiz-Martínez & Lavandero, 2015; Yang et al., 2017). To ensure synchronization and efficient pest control, it is thus essential that natural enemies arrive before their host or prey (Landis & Van Der Werf, 1997; Landis, Wratten & Gurr, 2000; Yang et al., 2017).

In perennial crops like fruit orchards, the early arrival of natural enemies was shown to be possible by the establishment of ground cover crops, *i.e.,* single plant species or a mix of plants sown in the inter-rows between fruit trees or growing spontaneous vegetation (Simoes et al., 2014; Gómez-Marco, Urbaneja & Tena, 2016; Bowers et al., 2020). Cover crops can increase the abundance of natural enemies (Aguilar-Fenollosa et al., 2011; Aguilar-Fenollosa & Jacas, 2013) and reduced pest populations (Dong et al., 2005; Irvin et al., 2006; Aguilar-Fenollosa et al., 2011; Gómez-Marco et al., 2016; Gómez-Marco, Urbaneja & Tena, 2016). For instance, in peach orchards, a cover crop of *Medicago sativa* L. (Fabaceae) increased predator densities, especially spiders, while reducing the population of leaf miner *Lyonetia clerkella* L. (Lepidoptera, Lyonetiidae) (Dong et al., 2005). However, few studies have evaluated the effect of intercropping on functional redundancies and niche complementarities among natural enemy guilds, as most of these studies have focused on single natural enemy guilds. In addition, these studies were performed during the growing season, and there are no studies on the effect of increasing populations of natural enemies in winter with alternative hosts to enhance their arrival and control pest populations in spring. Among the many pest species attacking plum orchards, aphids are the most important (Symmes et al., 2012; Cichon et al., 2013). They can cause direct as well as indirect damage, as they are the main viral disease vectors of the plum pox virus (Dragoyski, Stefanova & Kamenova, 2011) with resulting important economic losses (Cambra et al., 2006).

In this study, we investigated whether intercropping an oat cover within orchards before winter may induce the early arrival of natural enemies by providing alternative hosts and thus promote the biological control of aphid pests infesting plum orchards in spring compared to spontaneous vegetation. Previous studies have shown that aphid populations on cereals during the winter in Chile, as well as their natural enemies such as parasitoids and predators, are present (Ortiz-Martínez & Lavandero, 2018; Alfaro-Tapia et al., 2021). In addition, all aphid species attacking cereals and other wild graminaceous plants have not been observed to attack or damage plum trees shoots (Blackman & Eastop, 2000; Blackman & Eastop, 2007). However, plum aphids and aphids feeding in wild and cultivated Poaceae share similar natural enemies (Starý, 1995). Moreover, from our previous studies we can conclude that the main parasitoid species, *Aphidius platensis* (Hymenoptera, Braconiidae) of both plum and cereal aphids such as *Myzus persicae* and *Rhopalosiphum padi* (Hemiptera, Aphididae) respectively, are capable of shifting between aphid hosts (winter *vs.* spring host) (Alvarez-Baca et al., 2020). We hypothesize that (1) inter-cropped oat between plum trees attracts cereal aphids and their associated shared natural enemies during the winter, which leads to (2) an early arrival of shared natural enemy populations compared to the arrival of the main prevalent plum aphids in early spring. As a consequence, we expected (3) a lower aphid incidence on plum trees in the treatment with an oat cover crop, (4) due to a higher natural enemy abundance and parasitism rates on plum trees with no effect of hyperparasitism on the primary parasitoids.

## Materials & Methods

Field experiments were approved by ANID (Agencia Nacional de Investigación y Desarrollo- Chile) project number 1180601. Farms belonged to a single owner who willingly accepted to participate in the study and submitted their approval an allowance for using the field sites to FONDECYT- ANID (Fondo Nacional de Ciencia y Tecnología. Chile) project number 1180601 ANID biosecurity committee.

### Study area and experimental design

**Figure 1:**
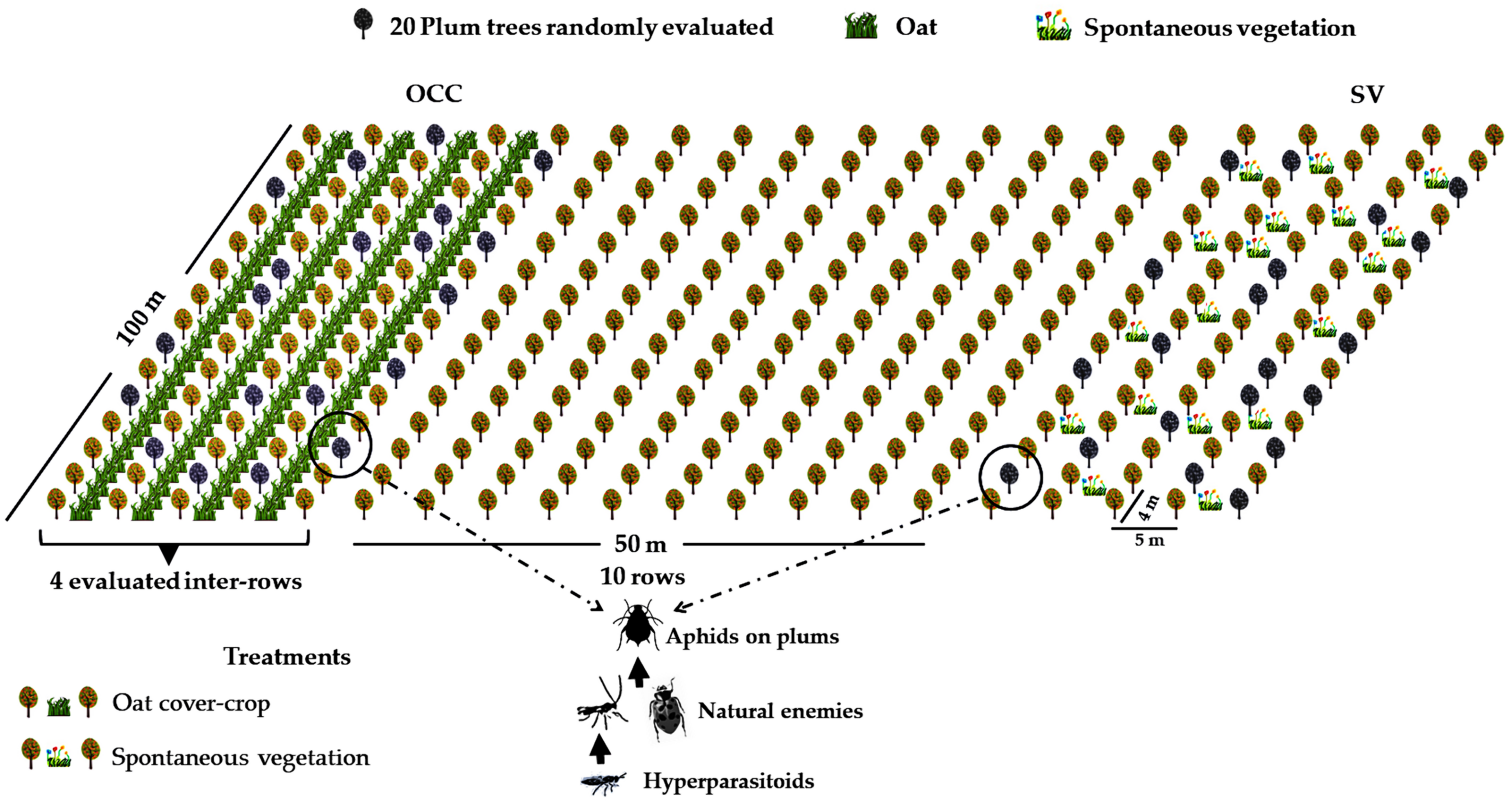
Field experimental setup. Treatment distribution in one of the evaluated fields (spontaneous vegetation (SV) and oat cover crop (OCC)). Plum trees, oats and spontaneous vegetation are shown. Gray trees represent the plum trees evaluated. Aphids, natural enemies and hyperparasitoids were evaluated in each treatment.

The study area was located at the district of Codegua, region of O’Higgins, in the Central Valley of Chile (34°08′S; 70°38′W). Four organic plum *Prunus domestica* L. (Rosaceae) cv. ‘D’Agen’ farms with the same rootstock and cultivar, with similar management and age structure (all orchards were planted from 2009 to 2013), were selected. Each farm was at least 10 ha. All orchards were managed following organic production guidelines, and neither synthetic pesticides nor fertilizers were used. The Central Valley of Chile is characterized by a temperate Mediterranean climate, with dry summers and mild, rainy winters (Sarricolea, Herrera-Ossandon & Meseguer-Ruiz, 2017). Temperatures vary from 25 to 35 °C in spring–summer (September–March) and between 3 to 13 °C in winter, with precipitation ranging from 22 to 130 mm during spring and from 300 to 900 mm in winter (June–August) (Montes et al., 2012; Directorate General of Civil Aviation of Chile, 2018). In each farm, two treatments were established with four replicates (each replicate consisted of a plot of 1 ha), resulting in a total of eight plots (see Table S1A for geographic coordinates of each plot). The treatments were as follow: the oat cover crop (OCC) treatment, consisting of four consecutive inter-rows of oat, *Avena sativa* L. (Poaceae) of at least 100 m long, with inter-rows sown during the second week of May in autumn, and the treatment without oat corresponding to four inter-rows with spontaneous vegetation (SV) (Fig. 1). Spontaneous vegetation rows consisted of the naturally occurring plants, which were periodically cut with a rotary cutter. Each tree row was separated by 5 m, and the space between plum trees along the row was 4 m (Fig. 1), with minimum, maximum and average distances between plots of 104.00 m, 665.58 m and 358.57 m respectively. (see Table S1B for more details). Tree management included regular mowing and pruning prior to the beginning of the flowering in the spring season. All four farms received similar management as they were all under the same company and certification guidelines. During spring, the SV treatment plots presented a patchily distributed presence of weeds such as *Malva* spp*.*, *Anoda hastata* Cav. (Malvaceae), *Taraxacum officinale* L. (Asteraceae) and graminaceous species such as *Poa annua* L., *Lolium* spp. (Poaceae) (see also Table S2). Both treatments in each plum plot were established at least 10 rows away (about 50 m) from each other to avoid interaction between treatments.

### Insect sampling

Aphids and their natural enemies were sampled over eight sampling dates from July 2018 (winter) to the end of the plum growing in November 2018 (spring). One week later the oat was cut out. Three sampling sessions were performed during winter (1: July 10th, 2: August 8th and 3: September 9th) with a monthly interval between sampling dates to monitor the establishment of insects in the oat inter-rows. There were five sampling dates in spring (4: September 25th, 5: October 9th and 6: 23th, 7: November 6th and 8: 20th) with an interval of 15 days between sampling dates to accurately record aphid colonization and breakdown that normally occurs in plum orchards (González, 1989). Both the inter-rows and the plum trees were studied to record the aphids and their associated natural enemies.

#### Aphid and parasitoid sampling on plum trees

During the winter months, plum trees were sampled for aphid eggs and colonies to ensure that the arrival of the first aphids and parasitoids would be detected in the study site, as aphids may feed as early as flowers bloom on plum trees (Grechi et al., 2008; Dedryver, Le Ralec & Fabre, 2010). A total of 169 aphid species have been reported in Chile, 128 of them having been introduced and many of these constituting important agricultural pests (Nieto Nafría et al., 2016). Of these, a group of five species has been related to plum trees in Chile (see Nieto Nafría et al., 2016); however, the complete assembly of related parasitoids has never been studied.

Spring sampling was realized as follows: In each plot, 20 randomly selected trees were sampled by collecting all living and parasitized aphids (*i.e.,* mummies) (Colfer & Rosenheim, 2001) on 20 shoots/tree. Living aphids were kept in a 50 mL tube with plant material inside to provide them a food source. Then, samples were taken back to the laboratory, where they were separated and counted. Aphids were determined to species level, following taxonomic keys (Blackman & Eastop, 2000; Blackman & Eastop, 2007; Nieto Nafría et al., 2016). Aphid mummies collected on the field were individualized in 1.5 mL Eppendorf tubes (with a small hole in the tip to let air pass) until adult parasitoid emergence in the laboratory. They were maintained under controlled conditions in climatic chambers (20 ± 1 °C, 65 ± 10% RH and 16L: 8D). The emergence of parasitoids was checked once daily. In addition, as living aphids were potentially parasitized, they were kept on hydrated plum leaves until mummy formation under the conditions mentioned above. Once mummies were formed, they were also isolated in Eppendorf tubes until emergence. If there was no mummification after 10 days, the aphids were discarded, as the development time is around 6-8 days on this species (Zamani et al., 2007). After their emergence, parasitoids and hyperparasitoids were identified using taxonomic keys (Starý, 1995; Tomanovic et al., 2014). Parasitism and hyperparasitism (*i.e.,* secondary parasitoid developing at the expense of a primary parasitoid) (Finke & Denno, 2005) rates were calculated as incidence, considering the number of parasitized and hyperparasitized shoots from the collected shoots with aphids.

#### Aphid and parasitoid sampling on the inter-rows

The density of aphids is scarce during the winter months, making it very difficult to find aphid colonies; thus, the aphid abundance was recorded by randomly walking through the middle of the inter-rows in a transect of 100 m during a period of 40 min by two observers. For the OCC treatment, live aphids and mummies were collected on the oat plants in the inter-rows, and for the treatment SV aphids from spontaneous graminaceous plants found along the inter-rows were also collected. All the material collected was taken back to the laboratory, where the number of winged and apterous aphid adults, as well as aphid nymphs and mummies was assessed. Living potentially parasitized aphids were kept in similar conditions as those explained before, except that aphids were kept on 10 cm high wheat plantlets within small pots (*h* = 25 cm, Ø = 8 cm) until mummification under the same climatic conditions. Parasitism rates were calculated as the number of mummified aphids from the total number of collected aphids.

In order to determine the density of aphids (number of aphids/tiller) and the parasitism rates on the inter-rows during the spring, 5 sub-sampling points were randomly chosen per plot. In each sampling point, all living aphids and mummies were sampled on 20 oat tillers randomly selected in the same row selected for the plum tree sampling. All living aphids were kept in a 50 mL tube with plant material inside as a food source until they were transferred back to the laboratory. For the SV treatment, we looked specifically for batches of wild graminaceous plants to follow the same procedure as described above. Aphids collected from both treatments were established in wheat plants (*Triticum aestivum* L.) assuming they could be potentially parasitized and were kept in the same laboratory conditions explained above until mummification. Parasitism rates were calculated as previously explained. After aphids were transferred into wheat plants at the laboratory, aphid mortality in the SV treatment was around 40–50%, whereas the remaining aphid species were able to establish under laboratory conditions.

### Natural enemy abundances

During the spring, yellow pan traps as well as pitfall traps were placed under the plum trees in order to determine the abundance of the main natural enemy groups. Yellow pan traps consisted of a plastic container (Ø = 26 cm, *h* = 10 cm) placed above ground level containing a solution of 400 mL of water and a few drops of detergent in order to diminish the water surface tension (De La Poza et al., 2005; Cheli & Corley, 2010; Droege et al., 2010; Albajes et al., 2013). They were used to sample populations of coccinellids (adults and larvae) (Coleoptera, Coccinellidae), syrphid flies (hoverflies) (Diptera, Syrphidae) and adult aphid parasitoids (Hymenoptera, Braconiidae). The pitfall traps consisted of a plastic cup of (Ø = 15 cm, *h* = 20 cm) buried at ground level, containing the same solution as mentioned above to collect carabid beetles (Coleoptera, Carabidae) and spiders (Araneae). Three yellow pan traps and three pitfall traps were placed per treatment per replicate (12 in total per treatment per trap type), with a 50m distance between traps as in Raymond, Ortiz-Martínez & Lavandero (2015). Sweep–net strokes were also used to collect flying individuals: coccinellid beetles, hoverflies and adult aphid parasitoids following a 20 m transect on each treatment (100 net strokes/transect) (Kogan & Pitre, 1980). All traps were opened for 15 days, after which trapped individuals were collected. Traps were then cleaned and filled with a new water solution. For all the types of traps, the collected specimens were individualized in 1.5 mL plastic tubes containing 95% alcohol, then they were counted and morphologically identified at the family level (Triplehorn & Johnson, 2005). The groups of natural enemies included in the analysis were: coccinellid beetles, hoverflies, adult aphid parasitoids sampled in the field (parasitoids emerged from aphids and mummies collected in the field were not included in this analysis), carabid beetles and spiders. For the analysis, the total number of individuals collected from each trap type (pan traps, pitfall traps and net strokes) was joined as a single value per plot (*N* = 4) per sampling date. These data were used to calculate the abundance of each group of natural enemies as well as the total natural enemy abundance.

### Statistical analyses

For the winter data, no statistical analysis was performed for the inter-rows, as the sampling effort on the inter-rows between the two treatment plots was not comparable in terms of abundance. For this reason, we determined the date of appearance and the presence/absence of the aphids, parasitoids and hyperparasitoids from the beginning of the winter until the end of the sampling in spring. Moreover, although absolute abundances could not be compared, we still established different intervals of abundances (1–100, 101–500 and >500) individuals per treatment on each group of insects found to have a general description of their dynamics over the time.

In order to assess the spatial autocorrelation of the aphid incidence as a function of distance between study sites (Diggle, Tawn & Moyeed, 1998; Diniz-Filho, Bini & Hawkins, 2003), the Moran’s Index (Moran, 1950) was calculated using the total aphid incidence per farm (4 farms) and the geographical coordinates (longitude and latitude) of each farm using the spatial autocorrelation tool of the software ArcGis *v.* 10.8 (Esri, 2019). The H_0_ of the spatial autocorrelation analysis assumes a random distribution of the values. Positive values of Moran’s I indicate an aggregate distribution pattern whereas negative values show a trend towards dispersion. Z-score represents the standard variations, and the higher the Z values, the lower the *p* values. When *p* values < *0.05*, H_0_ is rejected. No spatial autocorrelation of aphid incidence across study sites was observed (Moran’s I index = −0.88, *p* = 0.28, z-score = −1.09) and aphids showed a random spatial distribution pattern.

During spring on the plum trees, GLMMs were performed using treatments (OCC and SV) and the sampling dates as fixed factors, and the identities of the trees nested within the farm were used as random factors. To avoid zero inflated distributions, the number of aphids per shoot was converted into a proportion of shoot with aphids per plum tree. The aphid incidence was calculated as the proportion of shoots with aphids from the shoots without aphids per plum tree. The incidence of parasitized aphids was calculated as the proportion of shoots containing mummies from the shoots infested with aphids (*i.e.,* at least one mummy found in the shoots with aphids). In addition, the incidence of hyperparasitized aphids was calculated as the proportion of shoots containing hyperparasitized mummies from the shoots containing only mummies parasitized by primary parasitoids. Five sampling dates were considered for the aphid incidence and incidence of parasitism, whereas three sampling dates were used for the incidence of hyperparasitism as on dates 4 and 5 no hyperparasitoid was found. Those three response variables were analyzed assuming a binomial distribution with a logit function for proportional data. Total natural enemy abundance and the abundance of each natural enemy group was analyzed assuming a negative binomial distribution for counting data (see additional information on Table S3 for the selected model).

All the analyses were performed using the R package 3.6.5 (R Core Team, 2019). Statistical models were fitted according to the structure of the data. Generalized mixed models (GLMMs) were conducted using the lme4 package (Bates et al., 2015). The Akaike information criteria (AIC) was used to compare the different models after performing an ANOVA type II in the car package following a step-wise regression method (Fox et al. 2016). *Post hoc* pairwise comparisons were carried out using Tukey tests, correcting for multiple comparisons with the single-step method using the Multcomp package (Hothorn, Bretz & Westfall, 2008).

## Results

During the winter, on the plum trees, we did not find any aphid eggs or any nymphs or adults. On the inter-rows, aphids, parasitoids and hyperparasitoids arrived from the first date of winter sampling (July 10th) and increased as the season progressed (Table 1). Aphids were present in the OCC treatment during all sampling dates. For the parasitoids, we observed a similar pattern, as they were present from sampling date 1 through all the dates as the season progressed, with a higher incidence in the OCC treatment. Hyperparasitoids appeared at the fifth sampling date in the OCC treatment and remained present until the end of spring, whereas in the SV treatment, they appeared earlier: once at the first sampling date and again at the fourth sampling date until the end of the season. In both treatments, their presence was scarcer when compared to that of parasitoids.

**Table 1:** Temporal presence/absence of aphids, parasitoids and hyperparasitoids sampled in the inter-rows on both spontaneous vegetation (SV) and oat cover crop (OCC) treatments from the beginning of the winter until the end of the sampling in spring 2018. Signs represent presence (+) or absence (–) of aphids, parasitoids and hyperparasitoids on each sampling date (dates 1–8). +: 1-100; ++: 101-500; +++: >500 individuals; –: absence.

Sampling date treatments	7/10 Date 1	8/08 Date 2	9/11 Date 3	9/25 Date 4	10/9 Date 5	10/23 Date 6	11/6 Date 7	11/20 Date 8
*Aphids*								
SV	+	++	++	+	++	++	++	+++
OCC	++	++	++	++	+++	+++	+++	++
*Parasitoids*								
SV	+	+	+	+	+	+	+	+
OCC	+	+	+	+	++	++	++	++
*Hyperparasitoids*								
SV	–	–	–	+	+	+	+	+
OCC	+	–	–	–	+	+	+	+

**Figure 2:**
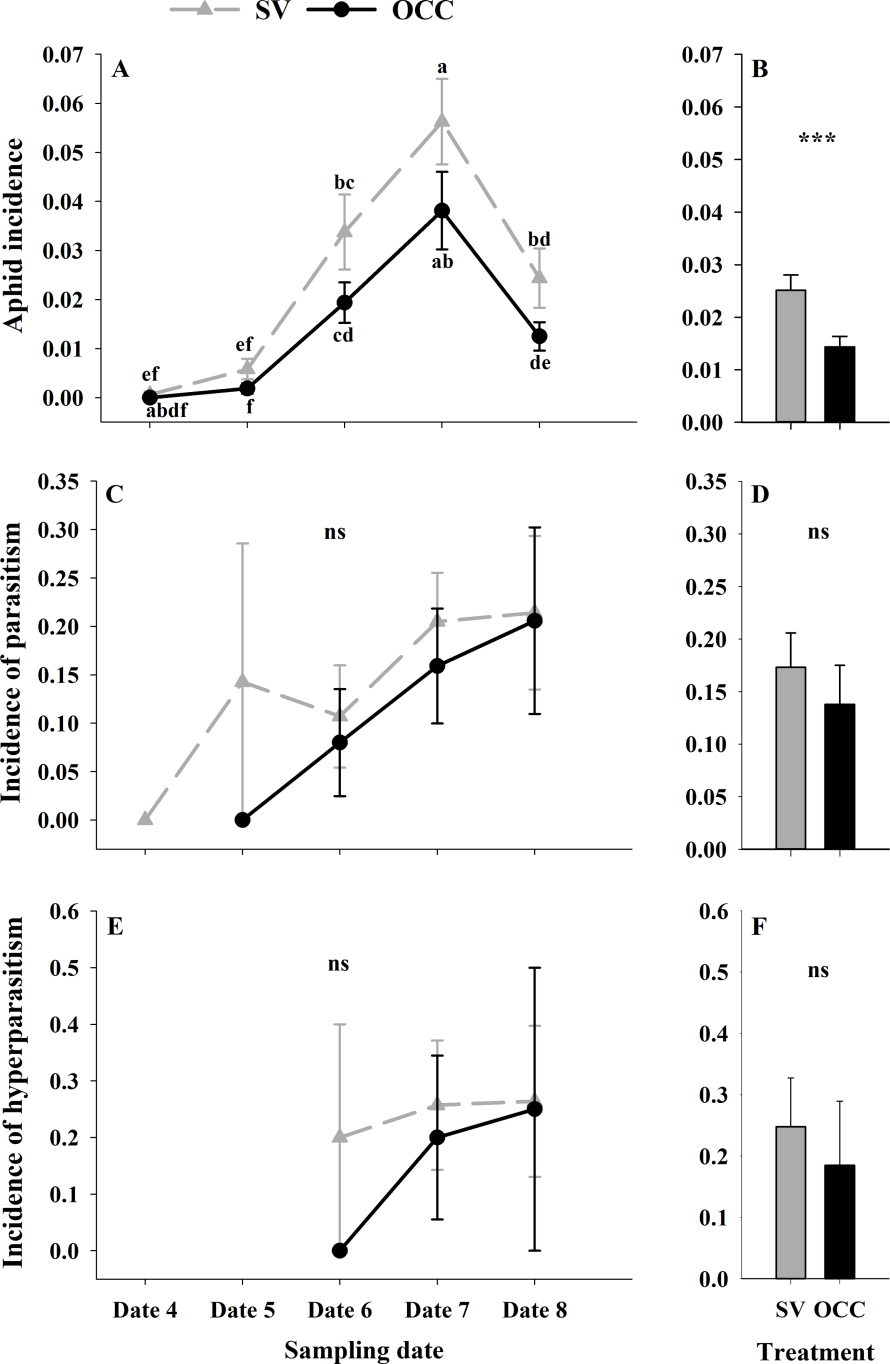
Seasonal variation of total aphids, parasitoids and hyperparasitoids incidence on plum trees. Seasonal variation during five sampling dates in spring 2018 and difference between treatments of total aphid, parasitoid and hyperparasitoid incidence on plums trees. Spontaneous vegetation SV (gray) and oat cover crop OCC (black). (A) Mean (± SE) proportion of shoots with aphids per sampling date; (B) mean (± SE) proportion of shoots with aphids per treatment; (C) mean (± SE) proportion of shoots with parasitized aphids per sampling date; (D) mean (± SE) proportion of shoots with parasitized aphids per treatment; (E) mean (± SE) proportion of shoots with hyperparasitized aphids per sampling date; and (F) mean (± SE) proportion of shoots with hyperparasitized aphids per treatment. Different letters indicate a significant difference between treatments and sampling dates and ‘ns’ non-significant differences. Tukey HSD *post hoc* tests (*P* < 0.05). Asterisks indicate significant differences: ‘***’ *P* < 0.001, ‘**’ *P* < 0.01 ‘*’ *P* < 0.05, ‘ns.’ non-significant *P* > 0.05. (Date 4: September 25th, date 5: October 9th, date 6: October 23th, date 7: November 6th and date 8: November 20th).

During the spring, a total of 4,865 aphids in the SV treatment were recorded, from which 76.81% corresponded to *Brachycaudus helichrysi*, 19.67% to *Aphis spiraecola* and 3.51% to *Myzus persicae*. Additionally, 2,752 aphids were collected in the OCC treatment; among these, 66.13% corresponded to *B. helichrysi*, 27.94% to *A. spiraecola* and 5.92% to *M. persicae*. The total aphid incidence was 3% in the SV treatment and 1% in the OCC treatment. The aphid incidence in plum trees differed per sampling date (GLMM: *χ*2 = 114.16, Df = 4, ***p* < 0.0001**) (Fig. 2A) and per treatment (GLMM: *χ*2 = 19.45, Df = 1, ***p* < 0.001**) (Fig. 2B), with more aphids in the SV treatment at all dates and the highest incidence of aphid infested shoots at the end of spring with no significant interactions (*p* = 0.87). There were no significant differences in the proportion of shoots with parasitized aphids (incidence of parasitism) between sampling dates (GLMM: *χ*2 = 7.50, Df = 4, *p* = 0.11) (Fig. 2C) or among treatments (GLMM: *χ*2 = 3.62, Df = 1, *p* = 0.06) (Fig. 2D). The proportion of shoots with parasitized aphids tended to increase in both treatments as the season went on until the last sampling date with a very low parasitism incidence (no interaction, *p* = 0.99). In addition, the incidence of hyperparasitism did not differ between sampling dates (GLMM: *χ*2 = 1.30, Df = 2, *p* = 0.52) (Fig. 2E) or between treatments (GLMM: *χ*2 = 0.47, Df = 1, *p* = 0.49) (Fig. 2F). Nevertheless, it tended to show a similar pattern to the incidence of parasitism, with a slight increase over the season in both treatments, with the highest incidence during the last sampling date (no interaction, *p* = 0.10).

**Figure 3:**
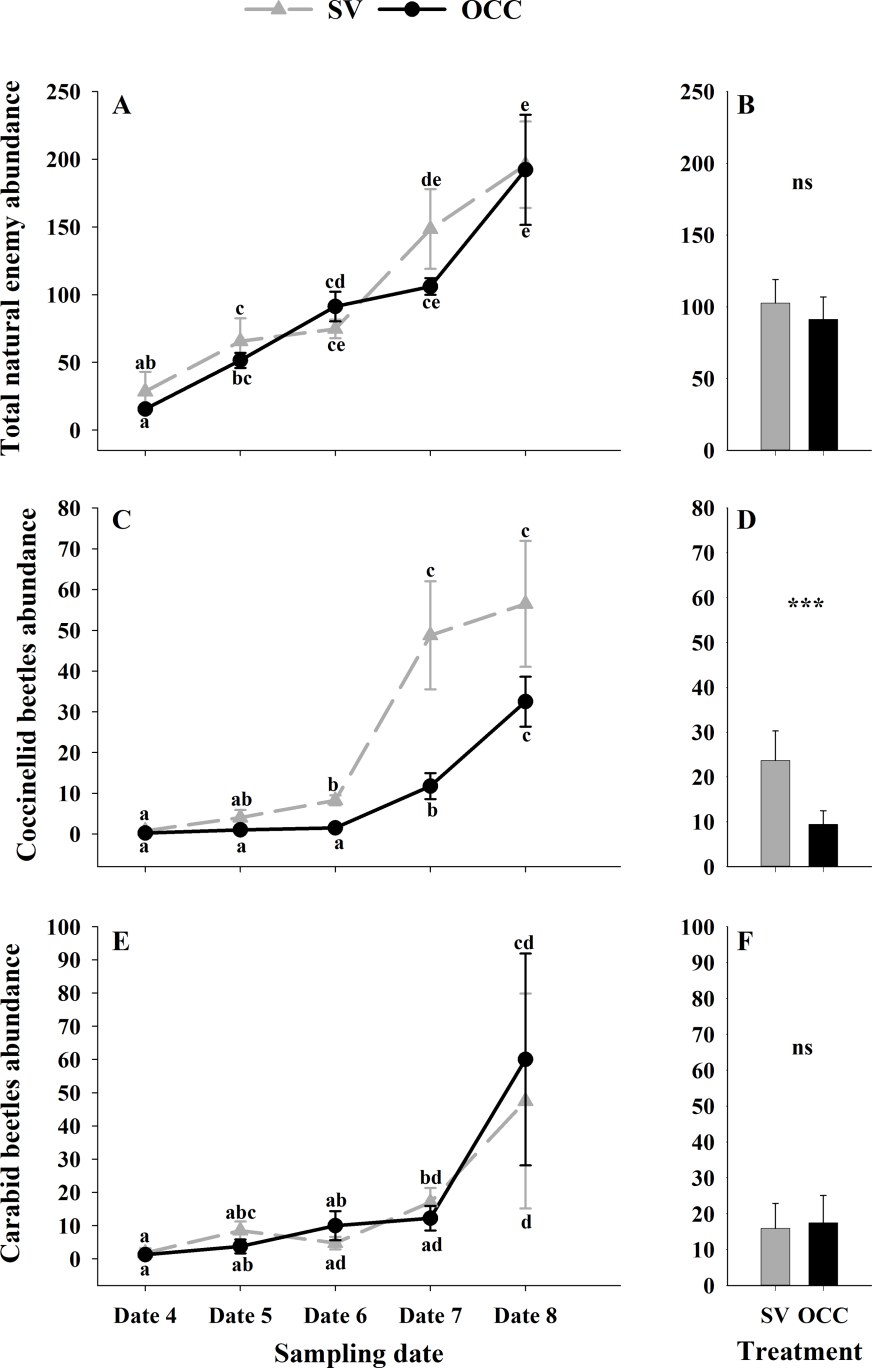
Seasonal abundance of the main natural enemies recorded on plum trees. Seasonal abundance during five sampling dates in spring 2018 and difference between treatments of the main natural enemies recorded in sampling traps. Spontaneous vegetation SV (gray) and oat cover crop OCC (black). (A) Mean (± SE) abundance of total natural enemies per sampling date; (B) mean (± SE) abundance of total natural enemies per treatment; (C) mean (± SE) abundance of coccinellids per sampling date; (D) mean (± SE) abundance of coccinellids per treatment; (E) mean (± SE) abundance of carabid beetles per sampling date; and (F) mean (± SE) abundance of carabid beetles per treatment. Different letters indicate a significant difference between treatments and sampling dates and ‘ns’ non-significant differences. Tukey HSD *post hoc* tests (*P* < 0.05). Asterisks indicate significant differences: ‘***’ *P* < 0.001, ‘**’ *P* < 0.01 ‘*’ *P* < 0.05, ‘ns.’ non-significant *P* > 0.05. (Date 4: September 25th, date 5: October 9th, date 6: October 23th, date 7: November 6th and date 8: November 20th).

**Figure 4:**
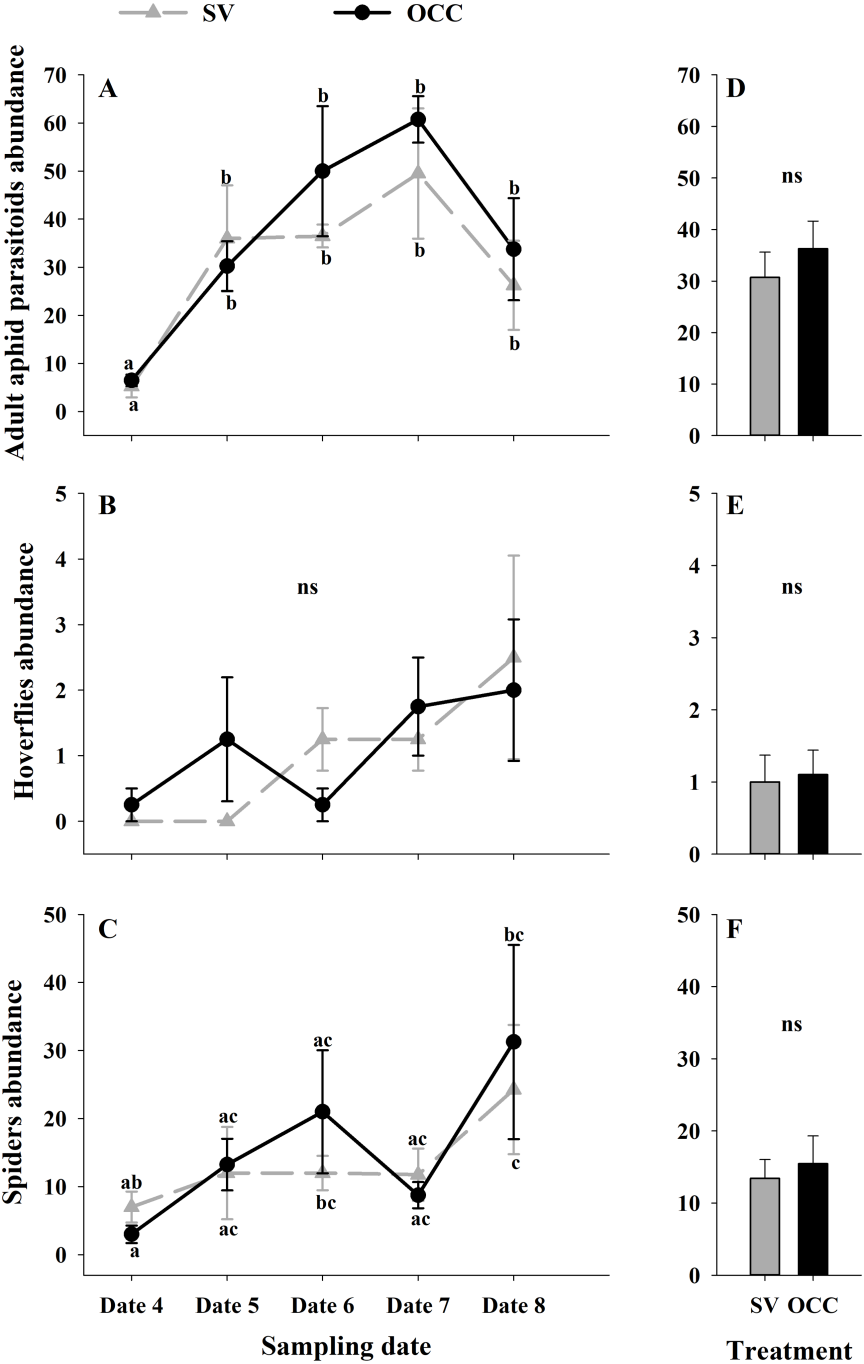
Seasonal variation of natural enemies in traps. Seasonal abundances during five sampling dates in spring 2018 and difference between treatments of the main natural enemies recorded in sampling traps. Spontaneous vegetation SV (gray) and oat cover crop OCC (black). (A) Mean (± SE) abundance of adult aphid parasitoids per sampling date; (B) mean (± SE) abundance of adult aphid parasitoids per treatment; (C) mean (± SE) abundance of hoverflies per sampling date; (D) mean (± SE) abundance of hoverflies per treatment; (E) mean (± SE) abundance of spiders per sampling date; and (F) mean (± SE) abundance of spiders per treatment. Different letters indicate a significant difference between treatments and sampling dates and ‘ns’ non-significant differences. Tukey HSD *post hoc* tests (*P* < 0.05). Asterisks indicate significant differences: ‘***’ *P* < 0.001, ‘**’ *P* < 0.01 ‘*’ *P* < 0.05, ‘ns.’ non-significant *P* > 0.05. (Date 4: September 25th, date 5: October 9th, date 6: October 23th, date 7: November 6th and date 8: November 20th).

From all the natural enemies recorded, adult aphid parasitoids were the most abundant, followed by carabid beetles, coccinellid beetles, spiders and hoverflies (Table S4). For the total natural enemy abundance, there were differences among sampling dates (GLMM: *χ*2 = 156.99, Df = 4, *p* < 0.0001) (Fig. 3A) but not between treatments (GLMM: *χ*2 = 2.50, Df = 1, *p* = 0.11) (Fig. 3B), with an increase in the abundance over time in both treatments (no interaction *p* = 0.25). Nevertheless, coccinellid beetles increased over time (GLMM: *χ*2 = 172.27, Df = 4, *p* < 0.0001) (Fig. 3C) and were more abundant in SV than in OCC (*χ*2 = 29.10, Df = 1, *p* < 0.0001) (Fig. 3D). A higher abundance for the SV treatment was observed mainly on dates 7 and 8 compared to the OCC treatment, without any significant interactions (*p* = 0.14). For carabid beetles, we found differences in the sampling date (GLMM: *χ*2 = 61.76, Df = 4, *p* < 0.0001) (Fig. 3E) but not between the treatments (GLMM: *χ*2 = 0.05, Df = 1, *p* = 0.82) (Fig. 3F), with an important increase at the end of the season (last sampling date) (interaction, *p* = 0.46). For the adult aphid parasitoids, significant differences among sampling dates (GLMM: *χ*2 = 72.84, Df = 4, *p* < 0.0001) (Fig. 4A) but not between treatments (GLMM: *χ*2 = 0.98, Df = 1, *p* = 0.32) (Fig. 4B) were found, for which the highest abundance was during date 7, but this decreased for the last sampling date (no interaction, *p* = 0.87). In the case of the hoverflies, there were no differences between sampling dates (GLMM: *χ*2 = 5.83, Df = 4, *p* = 0.21) (interaction, *p* = 0.73) (Fig. 4C) or among treatments (GLMM: *χ*2 = 0.22, Df = 1, *p* = 0.64) (Fig. 4D). Finally, there were differences in the abundance of spiders among sampling dates (GLMM: *χ*2 = 28.82, Df = 4, *p* < 0.0001) (Fig. 4E), but this did not differ between treatments (GLMM: *χ*2 = 0.00, Df = 1, *p* = 0.96) (Fig. 4F). In both treatments, we observed the highest abundance at the end of the season (interaction, *p* = 0.29).

## Discussion

Our results showed that the OCC treatment attracted cereal aphids and their associated natural enemies during the winter as proposed by our first hypothesis, which leads to the early arrival of shared natural enemies prior to the arrival of the plum aphids (second hypothesis). In concordance with our third hypothesis, we found a lower aphid incidence in the OCC treatment compared to the SV treatment on the plum trees. However, this was not due to a higher parasitism incidence or higher abundances of natural enemies. Moreover, no effect on the fourth trophic level was found, as we have no evidence for greater hyperparasitism of aphid colonies on plum trees in any of the two treatments (fourth hypothesis). Although the results provide evidence that the oat inter-row can be beneficial to decrease aphid densities, this is not due to an increase of activity or abundance of natural enemies.

During winter, we ensured the early arrival of aphid parasitoids on the oat inter-rows before the arrival of the target pest early in spring. Cover crops are used as overwintering resources and can harbor alternative host species for aphid parasitoids during a considerable time span (Welch & Harwood, 2014). Assuming that parasitoids on the cover crops will eventually move from the cover crop to the adjacent crop plants (Landis, Wratten & Gurr, 2000; Welch & Harwood, 2014), this would therefore increase biological control avoiding pest outbreaks (*e.g.*, aphids damaging fruit crop shoots). During spring, there were more parasitoids in the inter-rows in the OCC treatment, which acted as a great source of alternative hosts (cereal aphids) when compared to the SV treatment. However, even when the aphid incidence on plum trees was lower in the OCC treatment (56% less) compared to the SV treatment, this was a result of neither the parasitism rate nor the predator abundance (top-down effects). One possible explanation would be that the cereal aphid parasitoids do not switch in sufficient numbers to plum trees to control plum aphids, although in the laboratory this has been shown to be possible (Alvarez-Baca et al., 2020). It is also possible that parasitoids showed a greater preference for cereal aphids compared to plum aphids, being strongly influenced by the host from which they emerged (Morris & Fellowes, 2002).

When comparing the oat cover crop to spontaneous vegetation, the resource specialization hypothesis predicts that the increased plant diversity enhances the diversity of higher trophic levels by favoring species specialized on the additional resources (Hutchinson, 1959). If the spontaneous vegetation present is abundant and persistent (which was not the case for our study), greater pest control could be achieved as: (1) a more diverse vegetation would attract more diverse natural enemies (top-down effect) (Root, 1973; Poveda, Gómez & Martínez, 2008), and (2) a patchy/complex distributed area would reduce herbivore populations (Root, 1973). However, the OCC treatment, which had only one plant species (oat) but with a high plant density and coverage, and which was specifically sown to attract cereal aphids, showed a greater effect on aphid plum populations. It was showed that functionally diversified cover crops that increase functional diversity (attraction of parasitoids and predators that affect the target pest species) could enhance ecosystem services as pest control (Heemsbergen et al., 2004; Sattler et al., 2020). In our study system, the oat inter-row had not only a more homogeneous cover but also a higher functional value than the spontaneous vegetation. In the SV treatment, wild graminaceous plants, that can be attacked by cereal aphid hosts for target parasitoids, were reduced in proportion compared to introduced weeds from other plant families (Malvaceae, Asteraceae, Convolvulaceae). Moreover, the other weeds also did not sustain important populations of alternative hosts of the target natural enemies of this study (see results, Fig. 3).

In our study, two explanations can be provided to explain the absence of a link between the higher abundance of natural enemies and a decrease of aphid populations. The first one is the possible negative interactions between natural enemies, and the second one is linked to bottom-up effects. Negative interactions among natural enemies may have disrupting effects on pest suppression, interactions such as intraguild predation (*i.e.,* two predator species share the same prey species and could also feed on each other) (Polis, Myers & Holt, 1989; Lucas, 2005; Meisner et al., 2011; Traugott et al., 2012) and hyperparasitism (Finke & Denno, 2005; Jeavons et al., 2022). Our study would suggest that there is no relationship between the main predator abundances and the incidence of parasitism. Although coccinellid beetle abundance was higher in the SV treatment than in the OCC treatment, no measurable difference for the parasitism rates was found. However, we do not provide any direct evidence of intraguild predation or coincidental intraguild predation in this study; therefore, we cannot rule out this possibility. An increased abundance of the natural enemies present in SV treatment could be related to the increased abundance of their prey (Kandel, Tilmon & Shuster, 2016; Reznik et al., 2017). Likewise, hyperparasitoids are known to have negative impacts on parasitoid survival rates, with disrupting consequences on the population build-up of the primary parasitoid species affecting the outcome of biological control (Sullivan & Völkl, 1999; Tougeron & Tena, 2019). For instance, in Nagasaka, Takahasi & Okabayashi (2010), hyperparasitism rates on a group of Aphidiinae species, mainly *Aphidius colemani* Viereck (Hymenoptera, Braconidae) in sweet pepper and eggplant greenhouses using banker plants varied from 35% to 70% along the season, reducing the primary parasitism rates to less than 20% in one of the four years of sampling, with negative consequences on the control of aphids. By contrast, in our study, hyperparasitism did not seem to explain the absence of differences in parasitism rates in both treatments. These findings are in agreement with those of a study by Plećaš et al. (2014), where no differences of hyperparasitism rates (average of 10% or less) between simple and complex landscapes or between large and small-field landscapes were found, with no repercussion on the control of cereal aphids by parasitoids.

Another explanation is that the oat cover crops had a negative effect on the plum aphids through chemical and/or physical stimuli (bottom-up effects). During their host-plant selection process, herbivorous insects have to cope with different semiochemical cues from the plants as well as plant physical characteristics (*i.e.,* color, shape and texture) (Bernays & Chapman, 1994). In addition, cover crops may release chemicals that affect movement and feeding of aphids (Beizhou et al., 2011), for instance, there is previous evidence of *A. sativa* as a banker plant acting as a repellent on the same aphid species if pre colonized (Glinwood & Pettersson, 2000) and therefore could possibly also be repellent to other aphid species. They could also interfere with their ability to locate their host plant due to a lack of a clear olfactory stimuli resulting from the release of odor masking substances (Kennedy, Booth & Kershaw, 1961). Therefore, in our study, we speculate that oat in the OCC treatment might not allow plum aphids to reach plum trees because of odors emitted, making the crop difficult to be perceived by the herbivore, supporting the associational resistance hypothesis (Tahvanainen & Root, 1972), whereas in the case of the SV treatment the lesser vegetational density, as plants with available aphid hosts were less present between rows, supports the resource concentration hypothesis (Root, 1973). The pest density is reduced in a more diverse and patchy habitat (Root, 1973; Poveda, Gómez & Martínez, 2008), including a more diverse chemical habitat due to mixed volatiles from different plant species (Price et al., 1980).

On the other hand, cover crops, as well as providing refuge and alternative hosts, can be a sugar source for adult parasitoids through the provision of nectar and/or the honeydew produced by the aphids (Bugg & Waddington, 1994; Irvin et al., 2006; Vollhardt et al., 2010; Balzan, Bocci & Moonen, 2016; Luquet et al., 2021). In agroecosystems, nectar and honeydew are the most common available sources of sugar for natural enemies, especially parasitoids, which require non-prey food as part of their diet to increase their survival and fecundity (Wäckers, Van Rijn & Heimpel, 2008; Vollhardt et al., 2010). In our study, the OCC treatment clearly provided parasitoids a higher and constant source of honeydew compared to the SV. Previous studies have shown that honeydew can be as good as flower nectar in terms of quality (Vollhardt et al., 2010; Monticelli et al., 2020; Rand & Waters, 2020), as well as being the predominant source of sugar depending on the system (*e.g.*, Luquet et al., 2021). Therefore, the OCC treatment would offer more resources, such as sugar and alternative hosts, to parasitoids. The high coverage area of the oat through all the inter-row and the temporal availability suggest that parasitoids could possibly remain foraging in the cover crop without dispersing to the plum trees (Table 1). In contrast, the SV treatment, patchy distribution with less cover would offer fewer resources, forcing parasitoids to disperse. However, whether they disperse to the plum trees instead of other crop systems is not clear.

Even when both spontaneous vegetation and cover crops habitat management strategies are beneficial for biological control, we highlight the importance of focusing on a functional plant value instead of a taxonomic diversity (Gagic et al., 2015). Spontaneous vegetation can be easily promoted by farmers, does not require soil management, and can provide some functional plant diversity. However, low plant coverage and other problems such as the proliferation of invasive plant species or plants that act as a source of an insect pest could limit its usefulness (Venzon et al., 2019). In our study, although spontaneous vegetation did provide natural enemies, the decrease of plum aphid abundance was only achieved by the use of *A. sativa*. When carefully selected, inter cropping can offer many benefits such as: improving soil health by fixing nitrogen (Blanco-Canqui, Claassen & Presley, 2012), protecting soil from erosion, enhancing soil organic matter by providing a high biomass production (Isik et al., 2009; Nielsen et al., 2015), increasing water quality (Dabney, Delgado & Reeves, 2001), preventing weed plant growth (Brust, Claupein & Gerhards, 2014; Finney et al., 2017) by releasing growth inhibitors at high concentrations in the roots and shoots (Kato-Noguchi et al., 1994; Kato-Noguchi, Mizutani & Hasegawa, 1994). Specifically, there is evidence that *A. sativa* can have other benefits in addition to those previously mentioned such as: increasing mycorrhizal fungi populations and microbial biomass (Benitez, Taheri & Lehman, 2016), increasing earthworm populations compared to plots without cover crops (Roarty, Hackett & Schmidt, 2017). Moreover, as well as other cereals, oat is part of the banker plant system that provides alternative hosts for a parasitoid or predator of a target crop pest (Micha et al., 2000; Andorno & López, 2014) (for more beneficial examples, see Table S5). All of these ecosystem services, in addition to promote an early establishment of natural enemies, and/or to have repellent effects on the plum aphid populations, are key to develop a more sustainable approach.

## Conclusions

We can conclude that oat cover crops contribute positively to the control of aphid populations in plum orchards through an apparent bottom-up effect through physical and/or olfactive barriers for plum-associated aphids from the oat cover crop. In addition, it is important to mention that future studies should consider the effect of this or other cover crops on the assemblage of parasitoids and predators to fully understand the dynamics and consequences on aphid control and their natural enemies. Further studies on the species composition and food web structure in orchards with or without cover crops are needed to unravel the direct and indirect effects of these interactions on pest control.

##  Supplemental Information

10.7717/peerj.13299/supp-1 Supplemental Information 1 Raw data Aphid, parasitoid, hyperparasitoids, and predator main groups (carabid, spiders, hoverflies, parasitoids and coccinelids). Incidence per tree of aphid, parasitoid and hyperparasitoid. Predator data per traps.

10.7717/peerj.13299/supp-2 Supplemental Information 2 Geographic coordenates and distances among plots

10.7717/peerj.13299/supp-3 Supplemental Information 3 List of the most common weed species in the SV treatment found during the sampling

10.7717/peerj.13299/supp-4 Supplemental Information 4 Generalized linear mixed models (GLMMs) showing the full model evaluated for each response variable The models show the effect of the sampling dates and the treatments (SV and OCC) and the interaction between these two factors. For each variable, the chosen model, and for each level, the Chi-square statistical test (***χ***2), the degrees of freedom (df), and the *p*-value are represented.

10.7717/peerj.13299/supp-5 Supplemental Information 5 Number of natural enemy individuals collected per trap type per sampling date on two treatments: Spontaneous vegetation (SV) and Oat cover crop (OCC) Total numbers of each type of trap represent the sum of individuals per all the plots (4 plots) on each sampling date per treatment. Percentages represent the contribution of each type of trap on each group of natural enemies.

10.7717/peerj.13299/supp-6 Supplemental Information 6 Benefits of oat (*Avena sativa* L.) as a cover crop in different agricultural systems

## Additional Information and Declarations

### Field Study Permissions

The following information was supplied relating to field study approvals (i.e., approving body and any reference numbers): Field experiments were approved by ANID (Agencia Nacional de Investigación y Desarrollo- Chile) (No. 1180601). Farms belonged to a single owner who willingly accepted to participate in the study and submitted their approval an allowance for using the field sites to FONDECYT- ANID (Fondo Nacional de Ciencia y Tecnología. Chile).

### Data Availability

The following information was supplied regarding data availability: The raw data is available in the Supplemental File.
