# Supplementary material for: Effect of a cover crop on the aphid incidence is not explained by increased top-down regulation
Source: PeerJ. 2022 May 24;10:e13299. doi: 10.7717/peerj.13299 (PMC9138172; doi:10.7717/peerj.13299)
Supplement: Supplemental Information 2 [file peerj-10-13299-s002.docx]

**Table S1a.** Geographic coordinates of the sampling plots on plum fields during five sampling dates in spring 2018.

| **Plot** | **Coordinates** | | **Elevation (m)** |
| --- | --- | --- | --- |
|  | **Latitude** | **Longitude** |  |
| SV1 | 34°08′09.7″ S | 70°38′56.8″ W | 523 |
| SV2 | 34°08′12.7″ S | 70°38′49.3″ W | 527 |
| SV3 | 34°08′06.4″ S | 70°38′49.5″ W | 525 |
| SV4 | 34°08′22.3″ S | 70°38′38.7″ W | 530 |
| OCC1 | 34°08′10.4″ S | 70°39′00.7″ W | 524 |
| OCC2 | 34°08′17.1″ S | 70°38′50.8″ W | 527 |
| OCC3 | 34°08′05.9″ S | 70°38′41.6″ W | 523 |
| OCC4 | 34°08′22.5″ S | 70°38′46.1″ W | 540 |

**Table S1b.** Distances (m) between sampling plots during five sampling dates in spring 2018 in two treatments: Spontaneous vegetation SV and oat cover crop OCC. Maximum distance: 665.58m, minimum distance: 104m and average distance: 358.57m.

|  | **SV1** | **SV2** | **SV3** | **SV4** | **OCC1** | **OCC2** | **OCC3** | **OCC4** |
| --- | --- | --- | --- | --- | --- | --- | --- | --- |
| **SV1** |  |  |  |  |  |  |  |  |
| **SV2** | 215.3 |  |  |  |  |  |  |  |
| **SV3** | 211.15 | 196.64 |  |  |  |  |  |  |
| **SV4** | 600.8 | 397.43 | 560.29 |  |  |  |  |  |
| **OCC1** | 104 | 301.52 | 311.77 | 665.58 |  |  |  |  |
| **OCC2** | 271.08 | 128.7 | 332.31 | 344.13 | 325.95 |  |  |  |
| **OCC3** | 408.34 | 287.61 | 207.05 | 510.99 | 512.97 | 413.72 |  |  |
| **OCC4** | 477.33 | 314.25 | 503.36 | 192.29 | 525 | 200.62 | 519.83 |  |
