# Supplementary material for: Effect of a cover crop on the aphid incidence is not explained by increased top-down regulation
Source: PeerJ. 2022 May 24;10:e13299. doi: 10.7717/peerj.13299 (PMC9138172; doi:10.7717/peerj.13299)
Supplement: Supplemental Information 3 [file peerj-10-13299-s003.docx]

**Table S2**. List of the most common weed species in the SV treatment found during the sampling.

| **Species** | **Family** | **Local common name** |
| --- | --- | --- |
| *Helminthotheca echioides* | Asteraceae | Lengua de gato |
| *Hypochaeris radicata* | Asteraceae | Pasto del chancho |
| *Senecio vulgaris* | Asteraceae | Senecio |
| *Sonchus sp* | Asteraceae | Ñilhue |
| *Taraxacum officinale* | Asteraceae | Diente de león |
| *Brassicaceae spp.* | Brassicaceae | - |
| *Chenopodium album* | Chenopodiaceae | Quinguilla |
| *Convolvulus arvensis* | Convolvulaceae | Correhuela |
| *Anoda hastata* | Malvaceae | - |
| *Malva spp.* | Malvaceae | - |
| *Agrostis capillaris* | Poaceae | Chepica |
| *Avena fatua* | Poaceae | Avenilla |
| *Lolium spp* | Poaceae | - |
| *Poa annua* | Poaceae | Piojillo |
