# Supplementary material for: Effect of a cover crop on the aphid incidence is not explained by increased top-down regulation
Source: PeerJ. 2022 May 24;10:e13299. doi: 10.7717/peerj.13299 (PMC9138172; doi:10.7717/peerj.13299)
Supplement: Supplemental Information 4 — The models show the effect of the sampling dates and the treatments (SV and OCC) and the interaction between these two factors. For each variable, the chosen model, and for each level, the Chi-square statistical test (χ2), the degrees of freedom (df), and the p-value are represented. [file peerj-10-13299-s004.docx]

**Table S3.** Generalized linear mixed models (GLMMs) showing the full model evaluated for each response variables. The models show the effect of the sampling dates and the treatments (SV and OCC) and the interaction between these two factors. For each variable, the chosen model, and for each level, the Chi-square statistical test (**χ**2), the degrees of freedom (df), and the *p*-value are represented.

| **Variables** | **Selected model** | **Date** | | | **Treatment** | | | **Interaction** | | |
| --- | --- | --- | --- | --- | --- | --- | --- | --- | --- | --- |
|  |  | **df** | ***X^2^*** | ***p-value*** | **df** | ***X^2^*** | ***p-value*** | **df** | ***X^2^*** | ***p-value*** |
| Aphid incidence | model <- y ~ date*treatment+(1\|field/tree) | 4 | 114.16 | ***<2.2x10^−16^*** | 1 | 19.45 | ***<0.0001*** | 4 | 1.24 | 0.87 |
| Incidence of parasitism | model <- y ~ date*treatment +(1\|field/tree) | 4 | 7.50 | 0.11 | 1 | 3.62 | 0.06 | 3 | 0.10 | 0.99 |
| Incidence of hyperparasitism | model <- y ~ date*treatment + (1\|field/tree) | 2 | 1.30 | 0.52 | 1 | 0.47 | 0.49 | 2 | 0.00 | 0.10 |
| Total natural enemy abundance | model <- y ~ date*treatment + (1\|field) | 4 | 156.99 | ***<2x10^−16^*** | 1 | 2.50 | 0.11 | 4 | 5.41 | 0.25 |
| Coccinellid beetles abundance | model <- y ~ date*treatment +(1\|field) | 4 | 172.27 | ***<2.2x10^−16^*** | 1 | 29.10 | ***6.89x10^−8^*** | 4 | 6.90 | 0.14 |
| Carabid beetles abundance | model <- y ~ date*treatment + (1\|field) | 4 | 61.76 | ***1.24x10^−12^*** | 1 | 0.05 | 0.82 | 4 | 3.63 | 0.46 |
| Adult aphid parasitoids abundance | model <- y ~ date*treatment + (1\|field) | 4 | 72.84 | ***5.70x10^−15^*** | 1 | 0.98 | 0.32 | 4 | 1.22 | 0.87 |
| Hoverflies abundance | model <- y ~ date*treatment + (1\|field) | 4 | 5.83 | 0.21 | 1 | 0.22 | 0.64 | 4 | 2.04 | 0.73 |
| Spiders abundance | model <- y ~ date*treatment + (1\|field) | 4 | 28.82 | ***8.51x10^−6^*** | 1 | 0.00 | 0.96 | 4 | 4.96 | 0.29 |
