# Supplementary material for: Effect of a cover crop on the aphid incidence is not explained by increased top-down regulation
Source: PeerJ. 2022 May 24;10:e13299. doi: 10.7717/peerj.13299 (PMC9138172; doi:10.7717/peerj.13299)
Supplement: Supplemental Information 5 — Total numbers of each type of trap represent the sum of individuals per all the plots (4 plots) on each sampling date per treatment. Percentages represent the contribution of each type of trap on each group of natural enemies. [file peerj-10-13299-s005.docx]

| **Coccinellid beetles** | | | | | | | | | | | | | | | |
| --- | --- | --- | --- | --- | --- | --- | --- | --- | --- | --- | --- | --- | --- | --- | --- |
|  |  | **Date 4** | |  | **Date 5** | |  | **Date 6** | |  | **Date 7** | |  | **Date 8** | |
| **Treatment** | **Collecting method** | **N°** | **%** |  | **N°** | **%** |  | **N°** | **%** |  | **N°** | **%** |  | **N°** | **%** |
| SV | Yellow pantraps | - | - |  | 16 | 100 |  | 10 | 30.30 |  | 74 | 37.95 |  | 49 | 21.68 |
|  | Pitfall traps | - | - |  | 0 | 0 |  | 0 | 0.00 |  | 7 | 3.59 |  | 18 | 7.96 |
|  | Net-strokes | 3 | 100 |  | 0 | 0 |  | 23 | 69.70 |  | 114 | 58.46 |  | 159 | 70.35 |
|  | *Total* | 3 | 100 |  | 16 | 100 |  | 33 | 100 |  | 195 | 100 |  | 226 | 100 |
| OCC | Yellow pantraps | - | - |  | 1 | 25 |  | 1 | 16.67 |  | 23 | 48.94 |  | 24 | 18.46 |
|  | Pitfall traps | - | - |  | 2 | 50 |  | 0 | 0.00 |  | 6 | 12.77 |  | 15 | 11.54 |
|  | Net-strokes | 1 | 100 |  | 1 | 25 |  | 5 | 83.33 |  | 18 | 38.30 |  | 91 | 70.00 |
|  | *Total* | 1 | 100 |  | 4 | 100 |  | 6 | 100 |  | 47 | 100 |  | 130 | 100 |

**Table S4.** Number of natural enemy individuals collected per trap type per sampling date on two treatments: Spontaneous vegetation (SV) and Oat cover crop (OCC). Total numbers of each type of trap represent the sum of individuals per all the plots (4 plots) on each sampling date per treatment. Percentages represent the contribution of each type of trap on each group of natural enemies.

| **Carabid beetles** | | | | | | | | | | | | | | | |
| --- | --- | --- | --- | --- | --- | --- | --- | --- | --- | --- | --- | --- | --- | --- | --- |
|  |  | **Date 4** | |  | **Date 5** | |  | **Date 6** | |  | **Date 7** | |  | **Date 8** | |
| **Treatment** | **Collecting method** | *N°* | *%* |  | *N°* | *%* |  | *N°* | *%* |  | *N°* | *%* |  | *N°* | *%* |
| SV | Yellow pantraps | - | - |  | 5 | 14.71 |  | 1 | 5.26 |  | 15 | 22.06 |  | 12 | 6.32 |
|  | Pitfall traps | - | - |  | 21 | 61.76 |  | 16 | 84.21 |  | 43 | 63.24 |  | 174 | 91.58 |
|  | Net-strokes | 7 | 100 |  | 8 | 23.53 |  | 2 | 10.53 |  | 10 | 14.71 |  | 4 | 2.11 |
|  | ***Total*** | **7** | **100** |  | **34** | **100** |  | **19** | **100** |  | **68** | **100** |  | **190** | **100** |
| OCC | Yellow pantraps | - | - |  | 0 | 0 |  | 0 | 0.00 |  | 6 | 12.24 |  | 5 | 2.08 |
|  | Pitfall traps | - | - |  | 15 | 100 |  | 38 | 95.00 |  | 42 | 85.71 |  | 231 | 96.25 |
|  | Net-strokes | 5 | 100 |  | 0 | 0 |  | 2 | 5.00 |  | 1 | 2.04 |  | 4 | 1.67 |
|  | ***Total*** | **5** | **100** |  | **15** | **100** |  | **40** | **100** |  | **49** | **100** |  | **240** | **100** |

| **Adult aphid parasitoids** | | | | | | | | | | | | | | | | | | | | | | | | | | | | |
| --- | --- | --- | --- | --- | --- | --- | --- | --- | --- | --- | --- | --- | --- | --- | --- | --- | --- | --- | --- | --- | --- | --- | --- | --- | --- | --- | --- | --- |
|  |  | **Date 4** | |  | | **Date 5** | | | | |  | | **Date 6** | | | |  | | **Date 7** | | | |  | | **Date 8** | | | |
| **Treatment** | **Collecting method** | *N°* | *%* |  | | *N°* | | | *%* | |  | | *N°* | | *%* | |  | | *N°* | | *%* | |  | | *N°* | | *%* | |
| SV | Yellow pantraps | - | - |  | | 122 | | | 84.72 | |  | | 2 | | 1.37 | |  | | 12 | | 6.06 | |  | | 14 | | 13.33 | |
|  | Pitfall traps | - | - |  | | 0 | | | 0.00 | |  | | 0 | | 0.00 | |  | | 0 | | 0.00 | |  | | 0 | | 0.00 | |
|  | Net-strokes | 21 | 100 |  | | 22 | | | 15.28 | |  | | 144 | | 98.63 | |  | | 186 | | 93.94 | |  | | 91 | | 86.67 | |
|  | ***Total*** | **21** | **100** |  | | **144** | | | **100** | |  | | **146** | | **100** | |  | | **198** | | **100** | |  | | **105** | | **100** | |
| OCC | Yellow pantraps | - | - |  | | 108 | | | 89.26 | |  | | 2 | | 1.00 | |  | | 3 | | 1.23 | |  | | 6 | | 4.44 | |
|  | Pitfall traps | - | - |  | | 0 | | | 0.00 | |  | | 0 | | 0.00 | |  | | 0 | | 0.00 | |  | | 0 | | 0.00 | |
|  | Net-strokes | 26 | 100 |  | | 13 | | | 10.74 | |  | | 198 | | 99.00 | |  | | 240 | | 98.77 | |  | | 129 | | 95.56 | |
|  | ***Total*** | **26** | **100** |  | | **121** | | | **100** | |  | | **200** | | **100** | |  | | **243** | | **100** | |  | | **135** | | **100** | |
| **Hoverflies** | | | | | | | | | | | | | | | | | | | | | | | | | | | |  |
|  |  | **Date 4** | | |  | | **Date 5** | | |  | | **Date 6** | | | |  | | **Date 7** | | | |  | | **Date 8** | | | |  |
| **Treatment** | **Collecting method** | *N°* | *%* | |  | | *N°* | *%* | |  | | *N°* | | *%* | |  | | *N°* | | *%* | |  | | *N°* | | *%* | |  |
| SV | Yellow pantraps | - | - | |  | | 0 | 0.00 | |  | | 0 | | 0.00 | |  | | 0 | | 0.00 | |  | | 2 | | 20.00 | |  |
|  | Pitfall traps | - | - | |  | | 0 | 0.00 | |  | | 0 | | 0.00 | |  | | 0 | | 0.00 | |  | | 0 | | 0.00 | |  |
|  | Net-strokes | 0 | 0.00 | |  | | 0 | 0.00 | |  | | 5 | | 100 | |  | | 5 | | 100 | |  | | 8 | | 80.00 | |  |
|  | ***Total*** | **0** | **0** | |  | | **0** | **0** | |  | | **5** | | **100** | |  | | **5** | | **100** | |  | | **10** | | **100** | |  |
| OCC | Yellow pantraps | - | - | |  | | 0 | 0.00 | |  | | 0 | | 0.00 | |  | | 2 | | 28.57 | |  | | 3 | | 37.50 | |  |
|  | Pitfall traps | - | - | |  | | 0 | 0.00 | |  | | 0 | | 0.00 | |  | | 0 | | 0.00 | |  | | 0 | | 0.00 | |  |
|  | Net-strokes | 1 | 100 | |  | | 5 | 100 | |  | | 1 | | 100 | |  | | 5 | | 71.43 | |  | | 5 | | 62.50 | |  |
|  | ***Total*** | **1** | **100** | |  | | **5** | **100** | |  | | **1** | | **100** | |  | | **7** | | **100** | |  | | **8** | | **100** | |  |

| **Spiders** | | | | | | | | | | | | | | | |
| --- | --- | --- | --- | --- | --- | --- | --- | --- | --- | --- | --- | --- | --- | --- | --- |
|  |  | **Date 4** | |  | **Date 5** | |  | **Date 6** | |  | **Date 7** | |  | **Date 8** | |
| **Treatment** | **Collecting method** | *N°* | *%* |  | *N°* | *%* |  | *N°* | *%* |  | *N°* | *%* |  | *N°* | *%* |
| SV | Yellow pantraps | - | - |  | 17 | 35.42 |  | 4 | 8.33 |  | 8 | 17.02 |  | 6 | 6.19 |
|  | Pitfall traps | - | - |  | 18 | 37.50 |  | 17 | 35.42 |  | 13 | 27.66 |  | 32 | 32.99 |
|  | Net-strokes | 28 | 100 |  | 13 | 27.08 |  | 27 | 56.25 |  | 26 | 55.32 |  | 59 | 60.82 |
|  | ***Total*** | **28** | **100** |  | **48** | **100** |  | **48** | **100** |  | **47** | **100** |  | **97** | **100** |
| OCC | Yellow pantraps | - | - |  | 4 | 7.55 |  | 0 | 0.00 |  | 6 | 17.14 |  | 4 | 3.20 |
|  | Pitfall traps | - | - |  | 47 | 88.68 |  | 63 | 75.00 |  | 22 | 62.86 |  | 89 | 71.20 |
|  | Net-strokes | 12 | 100 |  | 2 | 3.77 |  | 21 | 25.00 |  | 7 | 20.00 |  | 32 | 25.60 |
|  | ***Total*** | **12** | **100** |  | **53** | **100** |  | **84** | **100** |  | **35** | **100** |  | **125** | **100** |
