# Supplementary material for: Effect of a cover crop on the aphid incidence is not explained by increased top-down regulation
Source: PeerJ. 2022 May 24;10:e13299. doi: 10.7717/peerj.13299 (PMC9138172; doi:10.7717/peerj.13299)
Supplement: Supplemental Information 6 [file peerj-10-13299-s006.doc]

| **Main crop** | **Cover crop** | **Benefit** | **Reference** |
| --- | --- | --- | --- |
| Wheat | **Oat,** pea, canola, vetch, radish and barley; mixture of 2, 3,6 species | Better soil properties | Ghimire et al., 2019 |
| - | **Oat**, white mustard, phacelia, ryegrass, rye, fodder radish | Preventing soil erosion by root density | De Baets et al., 2011 |
| Potato | **Oat**, triticale, rye,purple vetch, Egyptian clover and rapeseed and combitations. | Weed suppression, prevent soil erosion | Eshel et al., 2015 |
| Sweet corn | **Oat**, cereal rye, oilseed radish | Weed suppression, crop yield | O’Reilly et al., 2011 |
| Wheat | **Oat**, pea, canola,hairy vetch, forage radish and barley | Weed suppression and biomass production | Mesbah et al., 2019 |
| Organic pepper | **Oat,** ryegrass, rye, wheat, gelemen clover, egyptian clover,common vetch and hairy vetch | Weed suppression and biomass production | Isik et al., 2009 |
| Winter wheat in rotation with spring proso millet | **Oat,** rapeseed, flax, pea and a mixture with lentil,common vetch, berseem clover,barley, phacelia and safflower | Higher biomass production | Nielsen et al., 2015 |
| Tomato | **Oat**, rye, oilseed radish and mixtures | Higher profit margins | Belfry et al., 2017 |
| Winter wheat in rotation with corn | **Oat,** canola, radish, rye, pea, red clover, mixture of species | Weed suppression, biomass production, Nitrogen rentention | Finney et al., 2017 |
| Barley | **Oat**, ryegrass, phacelia, canola,rye, pea | Higher earthworm population | Roarty, Hackett & Schmidt, 2017 |
| Corn and soybean | **Oat** and rye | Reducing nitrate losses to surface waters | Kaspar et al., 2012 |
| - | **Oat,** clover,vetch and wheat | Increasing fungi populations and microbial diversity | Benitez, Taheri & Lehman, 2016 |
| Arugula and sweet pepper | **Oat** in a Banker plant system (oat – aphid – parasitoid) | Reducing populations of the pest aphid *M. persicae* in Arugula crop | Andorno & López, 2014 |
| Peach | **Oat** in a Banker plant system (oat – aphid – parasitoid) | Reducing populations of the pest aphid *M. persicae* | Mazzitelli, 2017 |
| Sweet pepper | **Oat** in a Banker plant system (oat – aphid – cecidomyd fly) | Reducing populations of the pest aphid *M. persicae* in greenhouses | Kuo‐sell, 1989 |

**Table S5.** Benefits of oat (*Avena sativa* L.) as a cover crop in different agricultural systems.

**References:**

Andorno AV, López SN. 2014. Biological control of *Myzus persicae* (Hemiptera: Aphididae) through banker plant system in protected crops. *Biological Control* 78:9–14. DOI: 10.1016/j.biocontrol.2014.07.003.

De Baets S, Poesen J, Meersmans J, Serlet L. 2011. Cover crops and their erosion-reducing effects during concentrated flow erosion. *Catena* 85:237–244. DOI: 10.1016/j.catena.2011.01.009.

Belfry KD, Trueman C, Vyn RJ, Loewen SA, Van Eerd LL. 2017. Winter cover crops on processing tomato yield, quality, pest pressure, nitrogen availability, and profit margins. *PLoS ONE* 12:1–17. DOI: 10.1371/journal.pone.0180500.

Benitez MS, Taheri WI, Lehman RM. 2016. Selection of fungi by candidate cover crops. *Applied Soil Ecology* 103:72–82. DOI: 10.1016/j.apsoil.2016.03.016.

Eshel G, Egozi R, Goldwasser Y, Kashti Y, Fine P, Hayut E, Kazukro H, Rubin B, Dar Z, Keisar O, DiSegni DM. 2015. Benefits of growing potatoes under cover crops in a Mediterranean climate. *Agriculture, Ecosystems and Environment* 211:1–9. DOI: 10.1016/j.agee.2015.05.002.

Finney DM, Murrell EG, White CM, Baraibar B, Barbercheck ME, Bradley BA, Cornelisse S, Hunter MC, Kaye JP, Mortensen DA, Mullen CA, Schipanski ME. 2017. Ecosystem Services and Disservices Are Bundled in Simple and Diverse Cover Cropping Systems. *Agricultural & Environmental Letters* 2:170033. DOI: 10.2134/ael2017.09.0033.

Ghimire R, Ghimire B, Mesbah AO, Sainju UM, Idowu OJ. 2019. Soil health response of cover crops in winter wheat–fallow system. *Agronomy Journal* 111:2108–2115. DOI: 10.2134/agronj2018.08.0492.

Isik D, Kaya E, Ngouajio M, Mennan H. 2009. Weed suppression in organic pepper (*Capsicum annuum L.*) with winter cover crops. *Crop Protection* 28:356–363. DOI: 10.1016/j.cropro.2008.12.002.

Kaspar TC, Jaynes DB, Parkin TB, Moorman TB, Singer JW. 2012. Effectiveness of oat and rye cover crops in reducing nitrate losses in drainage water. *Agricultural Water Management* 110:25–33. DOI: 10.1016/j.agwat.2012.03.010.

Kuo‐sell H ‐L. 1989. Getreideblattläuse als Grundlage zur biologischen Bekämpfung der Pfirsichblattlaus, *Myzus persicae* (Sulz.), mit *Aphidoletes aphidimyza* (Rond.) (Dipt., Cecidomyiidae) in Gewächshäusern. *Journal of Applied Entomology* 107:58–64. DOI: 10.1111/j.1439-0418.1989.tb00228.x.

Mazzitelli ME. 2017. Plantas refugio: su rol en el control biológico de *Myzus persicae*; Sulzer (Hemiptera: Aphididae) en el cultivo de duraznero *Prunus persica* (L.) Batsch (Rosaceae) en Junín, Mendoza, Argentina. Mendoza, Argentina: Universidad Nacional de La Plata. DOI: 10.35537/10915/64368.

Mesbah A, Nilahyane A, Ghimire B, Beck L, Ghimire R. 2019. Efficacy of cover crops on weed suppression, wheat yield, and water conservation in winter wheat–sorghum–fallow. *Crop Science* 59:1745–1752. DOI: 10.2135/cropsci2018.12.0753.

Nielsen DC, Lyon DJ, Hergert GW, Higgins RK, Holman JD. 2015. Cover crop biomass production and water use in the Central Great Plains. *Agronomy Journal* 107:2047–2058. DOI: 10.2134/agronj15.0186.

O’Reilly KA, Robinson DE, Vyn RJ, Van Eerd LL. 2011. Weed populations, sweet corn yield, and economics following fall cover crops. *Weed Technology* 25:374–384. DOI: 10.1614/wt-d-10-00051.1.

Roarty S, Hackett RA, Schmidt O. 2017. Earthworm populations in twelve cover crop and weed management combinations. *Applied Soil Ecology* 114:142–151. DOI: 10.1016/j.apsoil.2017.02.001.
